# Letermovir Prophylaxis and Cytomegalovirus Reactivation in Adult Hematopoietic Cell Transplant Recipients with and without Acute Graft Versus Host Disease

**DOI:** 10.3390/cancers13215572

**Published:** 2021-11-08

**Authors:** Delaney Wolfe, Qiuhong Zhao, Emma Siegel, Marcin Puto, Danielle Murphy, Julianna Roddy, Yvonne Efebera, Justin Tossey

**Affiliations:** 1 Comprehensive Cancer Center, James Cancer Hospital Solove Research Institute, The Ohio State University, 460 West 10th St., Columbus, OH 43210, USA; Qiuhong.Zhao@osumc.edu (Q.Z.); siegel.191@buckeyemail.osu.edu (E.S.); Marcin.puto@talaristx.com (M.P.); Danielle_murphy@rush.edu (D.M.); roddy35@osu.edu (J.R.); Yvonne.efebera@ohiohealth.com (Y.E.); Justin.tossey@osumc.edu (J.T.); 2 Franciscan Health Indianapolis, 8111 South Emerson Avenue, Indianapolis, IN 46237, USA; 3 Rush University Medical Center, 1620 W Harrison St., Chicago, IL 60612, USA; 4 OhioHealth Blood and Marrow Transplant, 3535 Olentangy River Road, Columbus, OH 43214, USA

**Keywords:** cytomegalovirus, graft versus host disease, allogeneic, letermovir, hematopoietic cell transplantation

## Abstract

**Simple Summary:**

Cytomegalovirus (CMV) infection and graft versus host disease (GVHD) both contribute to increased morbidity and mortality following allogeneic hematopoietic cell transplantation (allo-HCT). Since the development of GVHD can increase a patient’s risk of developing CMV infection post-allo-HCT, the aim of our retrospective study was to assess the effectiveness of letermovir prophylaxis in preventing CMV infection in these patients at high risk for CMV reactivation. Letermovir is an antiviral approved for the prevention of CMV infection. This study demonstrated that patients with GVHD had significantly fewer CMV infections when they received letermovir prophylaxis compared to patients who did not receive letermovir.

**Abstract:**

Cytomegalovirus (CMV) is the most clinically significant infection after allogeneic hematopoietic-cell transplantation (allo-HCT) and is associated with increased mortality. The risk for CMV reactivation increases with graft versus host disease (GVHD). GVHD contributes to significant morbidity and mortality and is treated with immunosuppressive therapies that can further increase CMV infection risk. Prophylaxis with letermovir, an oral antiviral approved to prevent CMV, has been shown to decrease the incidence of CMV infection post-allo-HCT in patients at high risk of CMV reactivation, but there is a lack of data confirming this benefit in patients with GVHD. In this single-center, retrospective study, we assessed the incidence of clinically significant CMV infection (CS-CMVi) in allo-HCT patients who received letermovir prophylaxis (*n* = 119) and who developed aGVHD compared to a control group (*n* = 143) who did not receive letermovir. Among aGVHD patients, letermovir prophylaxis decreased CS-CMVi in patients with aGVHD (HR 0.08 [95% CI 0.03–0.27], *p* < 0.001), reduced non-relapsed mortality (*p* = 0.04) and improved overall survival (*p* = 0.04). This data suggests that letermovir prophylaxis improves outcomes by preventing CS-CMVi in patients with aGVHD.

## 1. Introduction

Primary infection or reactivation of CMV occurs with highest incidence in CMV-seropositive patients undergoing allogeneic hematopoietic cell transplantation (allo-HCT), especially if they received stem cells from CMV-seronegative donors [1]. Patients with acute or chronic graft-versus-host disease (GVHD), patients with at least one human leukocyte antigen (HLA) mismatch, and patients with a haploidentical donor are also at an increased risk [1,2,3,4,5]. CMV reactivation occurs in 60% to 70% of CMV-seropositive patients, and primary infection affects 10% to 20% of CMV-seronegative recipients transplanted from CMV-seropositive donors [6,7,8,9]. While strategies of early detection and pre-emptive treatment have decreased the CMV disease incidence, CMV reactivation is still associated with significant morbidity and mortality and remains a significant factor associated with increased non-relapse mortality (NRM) after allo-HCT [10,11,12]. Additionally, agents used to treat CMV such as valganciclovir, ganciclovir, and foscarnet are associated with significant side effects such as nephrotoxicity and myelosuppression, which can be detrimental complications in patients post-allo-HCT [4].

Letermovir is an oral antiviral agent that inhibits the CMV-terminase complex to prevent CMV replication. Letermovir’s novel mechanism has no cross-resistance with other antiviral agents and has a favorable side effect profile, which makes it ideal for use in the prophylactic setting [13,14]. In controlled and retrospective trials, letermovir prophylaxis has demonstrated reduction in the incidence of CMV infection [15]. In a phase 3 randomized controlled trial of letermovir for CMV prophylaxis in allo-HCT patients, there was significantly less clinically significant CMV infection (CS-CMVi) in patients who received letermovir (37.5%) compared to placebo (60.6%) [11]. In a mortality analysis of the same phase 3 trial, among patients who developed CS-CMVi, the mortality rate was lower in patients who received letermovir (15.8%) compared to placebo (31%) [6]. Two retrospective studies also demonstrated reduced CS-CMVi or CMV viremia in patients who received letermovir compared to a control group [14,16]. While these studies included patients with GVHD, they were limited by a relatively small sample size and only a small portion of patients had received letermovir [14,15,16]. One small retrospective study found that extended letermovir administration beyond day 100 post allo-HCT is effective for CMV prophylaxis in patients with GVHD who require systemic immunosuppression, and that the presence of acute GVHD (aGVHD) grade ≥ 2 was associated with increased all-cause mortality [17].

Graft versus host disease is a serious but common complication associated with allo-HCT as a result of donor T-cell mediated stem cells that attack immunocompromised host tissues. Treatment of acute GVHD involves high dose corticosteroids, which suppress the immune system and put patients at even greater risk of infectious complications [5]. There is currently no standard of care for treating steroid-refractory acute GVHD, but treatment could include the newly approved ruxolitinib and/or additional immunosuppressive agents, which could contribute to increased infection risk [18]. Development of GVHD has been shown to increase the incidence of CMV reactivation, likely due to a prolonged state of immunosuppression [1,16,17]. Despite this increased risk, there is little published data focused on the efficacy of CMV prophylaxis for patients who develop aGVHD. Therefore, the goal of this study was to evaluate the efficacy of letermovir to prevent clinically significant CMV infection among allo-HCT patients who developed aGVHD.

## 2. Materials and Methods

This was a single-center retrospective cohort study of allo-HCT patients comparing the use of letermovir prophylaxis to a historical control group. Patients were included in this study if they were at least 18 years of age or older, CMV seropositive, and received an allo-HCT at The Ohio State University Comprehensive Cancer Center–James Cancer Hospital between 1 June 2016, through 30 June 2020. Letermovir use was incorporated into institutional infection prophylaxis guidelines at The Ohio State University for allo-HCT patients in July 2018. Therefore, patients who received allo-HCT from July 2018 through June 2020 comprised the letermovir group, while patients who received allo-HCT from June 2016 to July 2018 were included in the control group. This time frame encompasses a significant number of patients who received letermovir as standard of care and a comparable number in the control group who received an allo-HCT before letermovir was approved and widely used. Patients were followed through day +200 post-transplant.

The primary objective of this study was to determine the incidence of CS-CMVi within the first 200 days post-allo-HCT in patients who developed aGVHD grade ≥ 2. CS-CMVi was defined as CMV disease or CMV viremia leading to preemptive treatment with ganciclovir, valganciclovir, or foscarnet, as described previously [11]. Secondary outcomes included incidence of CS-CMVi in all patients, incidence of CMV viremia, duration of letermovir therapy, mortality, overall survival, and analysis of risk factors for the development of CS-CMVi. Acute GVHD was scored using the MAGIC Criteria [19].

Patient demographic and disease characteristics were summarized using descriptive statistics and compared using Wilcoxon rank sum test for continuous data and Pearson’s chi-squared test or Fisher’s exact test for categorical data, respectively. Time to CS-CMVi was calculated from the date of transplant to the initiation of preemptive treatment, and time to CMV viremia was calculated from the date of transplant to the first date of detectible CMV DNA. The cumulative incidence of CMV infection was estimated and compared using Gray’s test accounting for the competing risk of early death without CMV. The proportional sub-distribution hazards models were used to evaluate the associations between the use of letermovir and the risk of CMV adjusting for potential confounding factors. Non-relapse mortality (NRM) was defined as the time from the date of transplant to the date of death due to reasons other than relapse, treating relapse as a competing risk. NRM was analyzed similarly as described above for CMV outcome. Overall survival (OS) was calculated from the date of transplant to the date of death, censoring those alive at the date of last contact. The OS rate was estimated using the Kaplan–Meier method and Cox proportional hazard models were used to evaluate the associations between the use of letermovir and the risk of death. Letermovir was treated as a time-dependent covariate in all the models to account for letermovir’s impact on the risk of CMV only during the time period where patients were actually taking the drug. Stata 14 was used for the analyses and all the tests were two-sided with significance level at 0.05.

## 3. Results

Between 1 June 2016 and 30 June 2020, a total of 262 allo-HCT events were assessed with 119 events including patients who received letermovir prophylaxis and 143 events as part of the control group. Four of the included patients received two allo-HCTs within the study time frame, and in these patients, each transplant event was assessed separately. During the first allo-HCT for these four patients, letermovir was used in one allo-HCT event, and during the second allo-HCT, letermovir was used in three allo-HCTs. The two groups were similar with regard to diagnosis, donor class, stem cell source, donor positive CMV serostatus, conditioning regimen, and development of aGVHD grade ≥ 2 (Table 1). There were significant differences in age, gender, GVHD prophylaxis, and the use of ATG or a T-cell depleted graft (Table 1). Patients in the control group were older (*p* = 0.01), had more males (*p* = 0.048), had more ATG use or T-cell depleted grafts (*p* = 0.004), and less post-transplant cyclophosphamide (*p* = 0.003).

**Figure 1:**
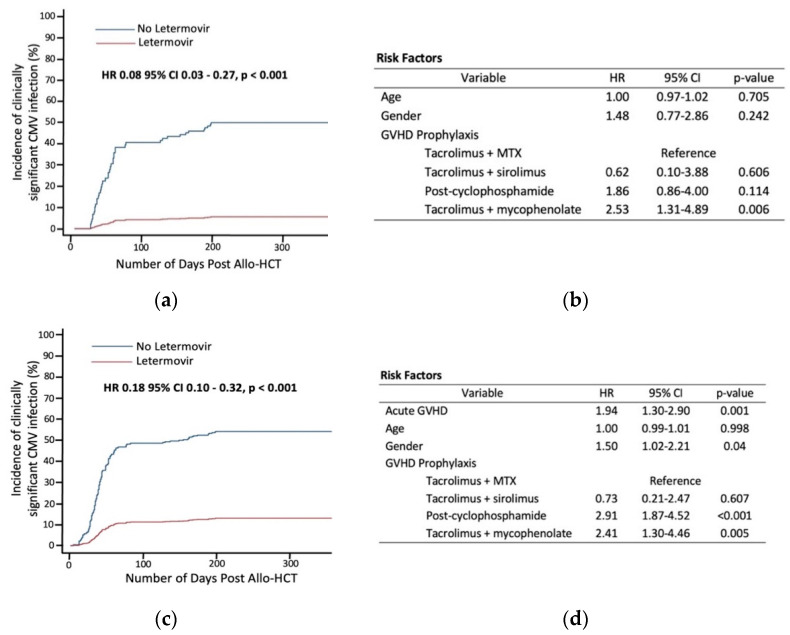
Incidence of clinically significant cytomegalovirus infection (CS-CMVi) in patients with and without letermovir and potential risk factors: (**a**) CS-CMVi in patients with acute graft versus host disease (aGVHD) grade ≥ 2; (**b**) Proportional sub-distribution hazards model of potential confounding factors in patients with aGVHD ≥ 2; (**c**) CS-CMVi in all patients; (**d**) Proportional sub-distribution hazards model of potential confounding factors in all patients.

Acute GVHD grade ≥ 2 developed in 69 patients (57.9%) in the letermovir group and 78 patients (54.5%) in the control group. Of the aGVHD patients, 26 (37.7%) in the letermovir group developed CS-CMVi compared to 71 patients (91%) in the control group. Among all transplant events, treatment for CS-CMVi was initiated in 110 allo-HCTs, which included 81 of 143 (56.6%) who did not receive letermovir compared to 29 of 119 (24.4%) who received letermovir (Table 2). The incidence of CS-CMVi was significantly reduced with the use of letermovir among patients with aGVHD grade ≥ 2 (HR 0.08 [95%CI 0.03–0.27], *p* < 0.001) within 200 days post-allo-HCT, adjusting for age, gender, GVHD prophylaxis and use of ATG or T-cell depleted graft (Figure 1a). A similar significant reduction in CS-CMVi was also seen in all patients within 200 days post-allo-HCT (HR 0.18 [95% CI 0.10–0.32], *p* < 0.001) (Figure 1c). No other variables assessed were associated with a decreased risk of CS-CMVi except letermovir (Figure 1b,d). Incidence of CMV viremia was lower in patients who received letermovir compared to those who did not receive letermovir (39.5% vs. 75.5%, *p* < 0.01; Table 2). The median peak CMV viremia was 770 IU/mL in patients who received letermovir compared to 1003 IU/mL in patients who did not receive letermovir (*p* = 0.03; Table 2).

In a multivariable analysis among all patients, letermovir reduced the risk of developing CS-CMVi, while the development of aGVHD and use of post-transplant cyclophosphamide for GVHD prophylaxis increased the risk of CS-CMVi (Figure 1d).

**Figure 2:**
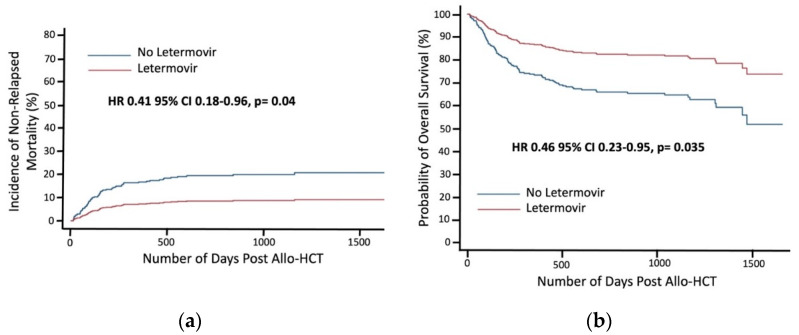
Survival outcomes in patients treated with or without letermovir prophylaxis: (**a**) Gray’s test was used to estimate non-relapsed mortality; (**b**) Kaplan-Meier method was used to estimate overall survival.

Non-relapsed mortality occurred in 8.4% of patients in the letermovir group compared to 9.7% of patients who did not receive letermovir (Table 2, Figure 2a). Overall survival was improved in patients who received letermovir (Figure 2b). Age and aGHVD were associated with increased risk of death (HR 1.03 [95% CI 1.02–1.05], *p* < 0.001 and HR 2.05 [95% CI 1.35–3.13], *p* = 0.001, respectively) and increased risk of NRM (HR 1.03 [95% CI 1.01–1.05], *p* = 0.012 and HR 3.59 [95% CI 2.01–6.40], *p* < 0.001, respectively).

The median duration of letermovir therapy was 95 days in all patients who received letermovir and 94 days in patients with acute GVHD grade ≥ 2. Nine patients were continued on letermovir indefinitely for recurrent CMV viremia. The most common reason for discontinuation of letermovir was due to completion of therapy at day +100 post-transplant.

## 4. Discussion

This single-center retrospective study showed that letermovir prophylaxis significantly reduced CS-CMVi in allo-HCT patients with aGVHD. Among patients with aGVHD, letermovir prophylaxis was the only variable that reduced CS-CMVi in a competing risk model. There was also significantly less CS-CMVi and CMV viremia in all patients who received letermovir prophylaxis. In addition, letermovir prophylaxis was also associated with significantly less risk of NRM as well as improved OS.

Current published literature has shown similar benefits with the use of letermovir for prevention of CS-CMVi, but our study adds additional analysis of patients who developed aGVHD post-transplant. The phase 3 trial of letermovir prophylaxis by Marty and colleagues included 373 patients who received letermovir and 192 patients who received placebo and found that 37.5% of patients who received letermovir developed CS-CMVi compared to 60.6% of patients who did not receive letermovir [11]. Anderson and colleagues conducted a retrospective analysis of 25 patients who received letermovir and 106 patients who did not that assessed the use of letermovir outside of a clinical trial setting. They reported that the cumulative incidence of CS-CMVi at 100 days post-allo-HCT was only 4% in patients who received letermovir compared to 59% who did not receive letermovir [14]. Studer and colleagues also published a retrospective analysis similar to Anderson and colleagues, but with a larger patient population of 432 patients [16]. This study found that 11.9% of patients who received letermovir developed CMV viremia compared to 24.6% who did not receive letermovir. Our study showed similar reduction in CS-CMVi in all patients with 24.4% who received letermovir and 56.6% in the control group developing CS-CMVi, which suggests that our data is likely generalizable. Our study also had relatively few exclusion criteria and therefore included a large number of both patients who received letermovir and those who did not, which strengthens the external validity of this data.

Most of the previously published studies did not report the outcomes of GVHD patients, and some only included a small number of these patients [6,11,14]. Although Studer and colleagues reported on a large GVHD population, this study was conducted in a time period prior to routine letermovir use, so only 42 of the 423 patients included in the study received letermovir [16]. In the mortality analysis of the phase 3 trial by Ljungman and colleagues, GVHD was associated with increased all-cause mortality, but there was no data reported about the effect of letermovir in these patients who developed GVHD [6]. While these studies have laid the foundation for the effect of letermovir on CS-CMVi rates, our study adds new information by focusing on the development of aGVHD and the incidence of CS-CMVi with and without letermovir prophylaxis.

Letermovir is approved for use up to day +100. Patients in our study were assessed over an extended time frame through day +200 post-transplant. This was intentional to capture the period of time after letermovir is typically discontinued in order to examine if the duration of letermovir was affected by CMV infection and/or the need for GVHD treatment. This study did not find any difference in the letermovir duration between patients who developed GVHD and those who did not. There were patients who received extended durations of letermovir with a range of up to 531 days of therapy. These extended durations occurred most commonly due to recurrent CMV viremia. The most appropriate duration of letermovir for patients who develop aGVHD is not well defined. It is possible that there would be benefit in continuing letermovir or considering reinitiation of letermovir while treating aGVHD. Further prospective trials are warranted to determine the extent of benefit of letermovir for CMV prophylaxis in patients who develop GVHD. We also showed that patients who received letermovir had improved OS and NRM. The improvement in OS is likely driven by the improvement in NRM. This is to be expected given that letermovir is an intervention that decreases infectious mortality.

The rates of aGVHD grade ≥ 2 reported in this study in both the letermovir group and control group (55.4% and 54.5%, respectively) are relatively high compared to averages previously published in the literature. The majority of our patient population (78%) received matched unrelated, mismatched unrelated, or haploidentical transplants, which are known to have a higher risk of GVHD compared to a matched related donor transplant [20,21]. Peripheral blood was the most common stem cell source in our patient population (77%), which also has an increased risk of GVHD compared to bone marrow or cord blood stem cell source [22]. Our high rate of aGVHD can likely be explained by the majority of our patient population having a high risk of developing aGVHD.

There are several limitations worth considering in this study. This was a retrospective chart review conducted at a single site, which inherently reduces generalizability and can be influenced by confounding factors. However, with minimal exclusion criteria, we were able to include a large sample size of patients and achieved results similar to multicenter trials, which suggests greater generalizability. There was an imbalance in gender between the two groups, which is unlikely to have impacted our results as there is no data to suggest a difference in outcomes, development of GVHD, or incidence of CS-CMVi based on gender. The median age of the control group was slightly older than the letermovir group (60 years and 56 years, respectively), which could have contributed to the lower mortality seen in the letermovir group as older patients have been shown to have a higher risk of mortality post-transplant. However, published literature has reported improved mortality rates with the use of letermovir regardless of age, which is consistent with our data and suggests that the age imbalance in our two groups is not clinically meaningful [11].

With inclusion of a historical control group, this group could have been influenced by changes in clinical practice over time. Changes in clinical practice during this study period included the decreased use of ATG and T-cell depleted graft and increased use of post-transplant cyclophosphamide for GVHD prophylaxis. Post-transplant cyclophosphamide has been shown to increase the incidence of CMV infection in CMV-seropositive recipients and was more commonly used in our letermovir group [23,24,25]. ATG has also been shown to be associated with increased risk of CMV reactivation, but it is important to consider that many of these trials including ATG were conducted before letermovir was used to prevent CMV infection [26,27,28]. ATG and T-cell depleted grafts were more commonly used in the historical control group. Given that there is a possibility for both ATG and post-cyclophosphamide to increase incidence of CMV infection, it is difficult to determine the net impact, if any, that these changes in practice may have had on the study results. However, if either the use of ATG/T-cell depleted graft or post-cyclophosphamide were to have a greater magnitude of increased risk for CMV, then that could have impacted our results by skewing the CMV infection rate in that study group. Prior studies have shown the cumulative incidence of CMV infection to be approximately 40% following allo-HCT with ATG/T-cell depleted graft or post-cyclophosphamide [25,28]. When looking at risk factors associated with CS-CMVi, GVHD prophylaxis with tacrolimus and mycophenolate appears to increase risk of CS-CMVi, but this result is likely skewed due to the small number of patients who received this prophylaxis regimen. Finally, this study was not designed to assess outpatient letermovir adherence.

## 5. Conclusions

Letermovir prophylaxis was effective at reducing the incidence of CS-CMVi among patients with aGVHD who are at an increased risk for CMV reactivation. Similar to other studies, letermovir prophylaxis also significantly reduced the incidence of CS-CMVi in all patients, the incidence of CMV viremia, and improved non-relapsed mortality and overall survival. This data suggests that letermovir prophylaxis should be considered in patients with aGVHD for the prevention of CMV infection.

## Figures and Tables

**Table 1:** Baseline characteristics.

	No Letermovir (*n* = 143)	Letermovir (*n* = 119)	*p*-Value
Age, median (range)	60 (18–76)	56 (21–74)	0.010
Male	86 (60.1)	57 (47.9)	0.048
Diagnosis			0.380
Acute Myeloid Leukemia	58 (40.6)	44 (37.0)	
Acute Lymphoblastic Leukemia	15 (10.5)	20 (16.8)	
Lymphoma (NHL and HL)	19 (13.3)	12 (10.1)	
Multiple Myeloma	2 (1.4)	5 (4.2)	
Myelodysplastic Syndrome/Myeloproliferative Neoplasms	37 (25.9)	23 (19.3)	
Chronic Myeloid Leukemia	2 (1.4)	2 (1.7)	
Chronic Lymphocytic Leukemia	6 (4.2)	6 (5.0)	
Other ^1^	4 (2.8)	7 (5.9)	
Donor class			0.656
HLA-mismatched unrelated HLA-matched unrelated	7 (4.9) 78 (54.5)	9 (7.6) 62 (52.1)	
HLA-matched related	34 (23.8)	24 (20.2)	
Haploidentical	24 (16.8)	24 (20.2)	
Stem Cell Source			0.239
Peripheral blood	115 (80.4)	86 (72.3)	
Bone marrow	26 (18.2)	32 (26.9)	
Cord blood	2 (1.4)	1 (0.8)	
Donor positive CMV serostatus ^2^	76 (53.1)	52 (43.7)	0.128
Conditioning Regimen			0.760
Myeloablative	70 (49.0)	56 (47.1)	
Reduced intensity	73 (51.0)	63 (52.9)	
GVHD Prophylaxis			0.003
Tacrolimus + methotrexate	94 (65.7)	67 (56.3)	
Tacrolimus + sirolimus	9 (6.3)	1 (0.8)	
Tacrolimus + mycophenolate	1 (0.7)	3 (2.5)	
Post-cyclophosphamide	36 (25.2)	48 (40.3)	
Other	3 (2.1)	0 (0.0)	
Use of ATG or T-cell depleted graft	54 (37.7)	25 (21.0)	0.004
Acute GVHD Grade			0.86
Grades 0–1	65 (45.5)	50 (42.0)	
Grade 2	54 (37.8)	48 (40.3)	
Grades 3–4	24 (16.8)	21 (17.7)	
Steroid dose ≥ 0.5 mg/kg ^3^	59 (75.6) (*n* = 78)	55 (79.7) (*n* = 69)	0.65

^1^ Including acute undifferentiated or mixed phenotype leukemia, blastic plasmacytoid dendritic cell neoplasm, eosinophilic leukemia, and T-cell prolymphocytic leukemia. ^2^ All patients had recipient positive CMV serostatus based on inclusion criteria. ^3^ Steroid dose reported for patients with acute GVHD grade ≥ 2.

**Table 2:** Results.

	No Letermovir (*n* = 143)	Letermovir (*n* = 119)	*p*-Value
CS-CMVi	81 (56.6)	29 (24.4)	<0.001
CMV viremia	108 (75.5)	47 (39.5)	<0.01
Peak CMV viremia in IU/mL, median (range)	1003 (51–81300)	770 (51–18178)	0.03
Mortality	62 (43.4)	38 (31.9)	0.06
Cause of Death			
Treatment-related	21 (14.6)	15 (12.6)	0.65
Non-relapsed	14 (9.7)	10 (8.4)	
Disease-related	27 (18.8)	13 (10.9)	

## Data Availability

The data presented in this study are available on request from the corresponding author.
